# Knowledge, attitudes, and practices related to breast cancer screening among female health care professionals: a cross sectional study

**DOI:** 10.1186/s12905-019-0819-x

**Published:** 2019-10-22

**Authors:** Humariya Heena, Sajid Durrani, Muhammad Riaz, Isamme AlFayyad, Rabeena Tabasim, Gazi Parvez, Amani Abu-Shaheen

**Affiliations:** 1 Research Center, King Fahad Medical City, Riyadh, Saudi Arabia; 2 Comprehensive Cancer Center, King Fahad Medical City, Riyadh, Saudi Arabia; 3 Department of statistics, University of Malakand, Chakdara, Pakistan; 4 Women’s Specialized Hospital, King Fahad Medical City, Riyadh, Saudi Arabia; 5 Department of Anaesthesia, Sheikh Khalifa Medical City, Ajman, United Arab Emirates

**Keywords:** Breast Cancer, Screening, Breast self-examination, Clinical breast examination, Mammography

## Abstract

**Background:**

Incidence of breast cancer in the Kingdom of Saudi Arabia (KSA) has increased in recent years. Screening helps in early detection of cancer and early diagnosis and timely treatment of breast cancer lead to a better prognosis. Women in the healthcare profession can have a positive impact on the attitudes, beliefs, and practices of general public. Therefore, it is important that the healthcare workers themselves have adequate knowledge and positive attitudes. We conducted a study to assess the knowledge, attitudes, and practices related to breast cancer screening among female healthcare professionals.

**Methods:**

A cross-sectional study was conducted on female health professional of KFMC (King Fahad Medical City). Data was collected using a pre-designed, tested, self-administered questionnaire. The questionnaire included specific sections to test the participants’ knowledge, attitude, and practices related to cervical cancer and its screening. Data analysis was done using descriptive statistics.

**Results:**

A total of 395 health care workers participated in this study. The mean age of the participants was 34.7 years. Participants included physicians (*n* = 63, 16.0%), nurses (*n* = 261, 66.1%), and allied health workers (*n* = 71, 18.0%). Only 6 (1.5%) participants had a good level of knowledge of breast cancer and 104 (26.8%) participants demonstrated a fair level of knowledge. Overall, 370 (93.7%), 339 (85.8%), and 368 (93.2%) participants had heard of breast self-examination, clinical breast examination, and mammography, respectively. A total of 295 (74.7%) participants reported practicing breast self-examination, 95 (24.1%) had undergone clinical breast examination, and 74 (18.7%) had ever undergone mammography.

**Conclusion:**

The knowledge, attitudes, and practices related to breast cancer screening were found to be lower than expected. Active steps are required to develop educational programs for the health care staff, which might empower them to spread the knowledge and positively influence the attitudes of female patients in the hospital.

## Background

Breast cancer is the most common cancer among women worldwide [1]. In 2018, over 2 million new cases of breast cancer were diagnosed globally accounting for 11.6% of all cancers. Breast cancer is also the most common cause of cancer-related deaths in women [1]. It is no longer prevalent only in the developed part of the world but is commonly reported in the developing countries as well. In Kingdom of Saudi Arabia incidence of breast cancer has been on the rise in recent years with number of cases increasing from 1152 per 100.000 inhabitants in 2008 to 1473 per 100.000 inhabitants in 2010, and 1826 per 100.000 inhabitants in 2014 [2, 3]. The KSA health council 2014 cancer registry reported breast cancer to be the most common cancer in women accounting to 28.7% of all cancers while another study attributed 13.08% of all deaths to breast cancer 98% of which occurred in women and 12% in men [3, 4].

Although there has been immense progress in the treatment of breast cancer, prognosis remains poor in developing countries including KSA [4]. An important reason for the poor prognosis could be a delay in diagnosis. When breast cancer is diagnosed at an early stage, prognosis is believed to be good with reduced morbidity and mortality [5]. Therefore, steps should be taken to ensure early detection and timely treatment. Two vital strategies for early detection include early diagnosis and screening [6]. An important aspect of early diagnosis includes increasing the awareness of early signs of cancer among physicians, nurses, other healthcare workers as well as the general population [7]. Screening, on the other hand, includes employing simple tests to identify individuals with cancer even before symptoms appear. Breast self-examination (BSE), clinical breast examination (CBE), and mammography are well recognized screening methods for breast cancer [6, 7]. Although in recent international guidelines, which focus on developed countries, the timeframes for screening have been questioned, this may not apply to the developing countries including Saudi Arabia where the awareness is very low and patients routinely present at advanced stage of breast cancer [8, 9].

Breast cancers in women from Arab populations have different characteristics and affected patients are at least a decade younger. Hence, the of Ministry of health in KSA guidelines in contrast to international guidelines recommend the use of screening strategies with mammography for the detection of breast cancer in women aged 40–49 years every 1 to 2 years. The indication that higher benefit on breast cancer mortality justifies a recommendation in favor of implementing breast cancer screening using mammography in this age group in this population.

Based on local cancer registry data, the incidence of breast cancer in the KSA for the age group 50–69 years is similar to the ones reported in the literature in other countries. Hence the Ministry suggests screening with mammography in women aged 50–69 years every 2 years and no screening with mammography for women aged 70–74 years, however a nationalized large scale screening program is yet to take off [10].

In KSA despite the healthcare facilities being free of cost, utilization of breast cancer screening methods, including mammography, is very low with one study reporting that out of women 50 years or older, 89% of them reported not having a clinical breast examination (CBE) and 92% of women reported never having a mammogram in the past year [11].

For effective screening and early diagnosis, adequate knowledge and awareness are of utmost importance. Women healthcare workers can bring about a significant change in the overall perspective of their female patients, regarding screening practices and positively influence their attitudes and beliefs [12]. They are also the first point of contact irrespective of their specialty of work for not only their female patients but also female relatives and friends for advice regarding breast cancer screening. Females usually feel embarrassed to talk about this issue with their male physicians. Consequently, measures are required to educate women and spread awareness. To achieve this, an important step would be to ensure that female healthcare professionals themselves possess adequate knowledge which they can transmit to their patients, relatives and acquaintances [13].

Several studies have been conducted in other developing countries to assess the knowledge and practices of breast cancer screening both in the general population as well as specifically in healthcare professionals [14–18]. In KSA, also several similar studies have been conducted on the general population [19]. However, the number of studies conducted on healthcare professionals in KSA have been limited. We, therefore, conducted this study to assess the knowledge, attitudes, and practices related to breast cancer screening among female healthcare professionals.

## Methods

### Study design and study population

A cross-sectional study was conducted on female healthcare workers (with at least 1 year of clinical experience) in 2018, including physicians, nurses, and allied health staff, at King Fahad Medical City (KFMC), Riyadh, Saudi Arabia.

### Data collection

Data were collected using a pre-designed, pre-tested, and self-administered questionnaire. The questionnaire was developed from previous studies after an in depth literature review [14–18, 20–22]. Before administering the questionnaire to the study population, the face validity of the questionnaire was ensured by a committee of experts in research methodology, obstetrics and gynecology, and oncology. A pilot study was conducted on 70 participants to ensure the clarity and reliability of the questionnaire. Cronbach’s alpha was used to evaluate the reliability which was found to be > 0.70. A trained research assistant randomly approached the subjects in each department and distributed the questionnaires. A survey cover sheet explaining the study was attached to the questionnaire for the participants to sign and complete. Complete anonymity was maintained to protect participants’ identity and to ensure confidentiality of data.

After an extensive literature search, the various survey questions were formulated and the questionnaire was divided into several sections. Some of the question were modified or deleted as per the recommendations of the expert committee since they were either off topic or not suitable for health care workers. The questionnaire included different parts.

First part elicited socio-demographic data on age, clinical experience, education, designation, department, marital status, age at marriage, number of pregnancies, number of children, history of breast cancer, and family history of breast cancer of each study participant.

Questions relating to knowledge of breast cancer were included in the second part. These questions were included under three categories: potential risk factors, signs and symptoms, and ways of screening/diagnosis of breast cancer including BSE, CBE, and mammography.

The respondents were requested to record their answers by choosing one of the three options: ‘Yes’, ‘No’, or ‘Don’t Know’. The scale was then dichotomized (Yes = 1 and No/Don’t Know = 0) and the total knowledge score for each participant was computed by adding up (maximum score of 30). The total score was then categorized as poor knowledge (score of 0–4), fair knowledge (score of 5–14), and good knowledge (score of 15–30).

Participants’ attitude regarding breast cancer was assessed in the third part by asking them to rate 10 specific statements on a 5-point Likert scale. Following 10 statements were included in the questionnaire: 1) Any woman is at risk for breast cancer; 2) Breast cancer can be prevented; 3) If I examine my breast myself, I cannot detect abnormalities in my breast; 4) There is no reason to examine my breasts; 5) If I knew the benefit of breast self-examination, I would have done it by now; 6) Women prefer female doctor for breast examination; 7) If there is no problem in the breasts, periodic breast examinations by a physician are not required; 8) Early detection methods have no effect on treatment; 9) Personal hygiene decreases breast cancer risk; 10) By early diagnosis of breast cancer, the person will have prolonged life. Participants were asked to choose one of the following options for each of the statements above: ‘strongly agree’, ‘agree’, ‘neither agree nor disagree’, ‘disagree’, or ‘strongly disagree’. For presenting results, ‘strongly agree’ and ‘agree’ were combined; similarly, ‘disagree’ and ‘strongly disagree’ were combined.

Participants’ practices were assessed through the last section asking specific questions about BSE, CBE, and mammography. Participants were asked whether they had heard of BSE, CBE, and mammography and whether they believed these tests were useful for early detection of breast cancer. Other questions under BSE enquired whether they had been taught BSE, whether they practice BSE, what age should BSE be done, how frequently should BSE be done, what is the best time to do BSE, what action must be taken when any abnormality is found in BSE, and what, according to them, are the benefits of BSE. Similarly, questions under CBE sought information on whether they had undergone CBE, how CBE is done (by whom, using what), and how often should CBE be done. Questions on mammography tested the participants’ knowledge on what age mammography should be started, how often should it be done, and whether they had undergone mammography.

### Ethical considerations

An informed consent was obtained from each participant before enrolment and no compensation or incentive was paid to the participants for this study. The study was approved by the ethics committee at KFMC.

### Sample size estimate

The study population was stratified according to their professions into three groups: physicians, nurses, and allied healthcare workers. To ensure appropriate representation from each group of healthcare professionals, the proportionate population sampling method in the form of 4:1:1 for nurses, physicians, and allied healthcare workers, respectively, was adopted. Hence, 260 nurses (out of 2400), 65 physicians (out of 600) and 65 allied health care workers (out of 700) were approached on a random basis from each department and the total sample size was determined to be 390.

### Statistical analysis

The statistical package for social science (IBM SPSS statistics 22. Ink) was used for data analysis. Descriptive statistics (i.e., frequencies, percentages, mean [standard deviation, SD] /median [interquartile range, IQR]) were used to describe the demographic characteristics, knowledge, attitude, and practice of breast cancer screening.

## Results

### Socio-demographic characteristics

A total of 420 questionnaires were distributed to the KFMC female employees of which 395 (94%) were returned and included in the analysis. The mean age (SD) of the participants was 34.7 (8.3) years. Majority of the participants were married (*n* = 239, 60.5%). Respondents included 261 (66.1%) nurses, 63 (16.0%) physicians, and 71 (18.0%) other healthcare workers including pharmacists, dieticians, technicians, health educators, physiotherapists, and therapists. Majority of the participants were bachelor’s degree holders (*n* = 272, 68.9%) and 52 (13.2%) had a postgraduate qualification. Average work experience was 10 [6–16] years. Nine (2.3%) participants reported having history of breast cancer and 40 (10.1%) participants reported having a first-degree relative with history of breast cancer (Table 1).

**Table 1:** Participants’ Socio-Demographic Characteristics

Variables	Mean (SD)/ Median (IQR)^a^
Age^c^	34.7 (8.3)
Experience in years^c^	10 (6–16)
Age at marriage^c^(*n* = 252)	26.2 (4.4)
Number of pregnancies^c^ (*n* = 252)	2 (1–3)
Designation:	n (%)^b^
Physician	63 (16.0)
Nurse	261 (66.1)
Pharmacist	5 (1.3)
Dietician	3 (0.8)
Technician	23 (5.8)
Health educator	1 (0.3)
Physiotherapists	7 (1.8)
Therapist	21 (5.3)
Others	11 (2.8)
Hospital/Center/department^c^:	
Comprehensive Cancer Center	16 (4.1)
National Neurosciences Institute	9 (2.3)
King Salman Heart Center	19 (4.8)
Obesity Endocrine and Metabolic Center	1 (0.3)
Women’s Specialized Hospital	49 (12.4)
Children’s Specialized Hospital	135 (34.2)
Rehabilitation Hospital	33 (8.4)
Main Hospital	101 (25.6)
Others	28 (7.1)
Level of Education^c^:	
High School or Diploma	69 (17.5)
Bachelor	272 (68.9)
Master or PhD	52 (13.2)
Marital Status^c^:	
Single	143 (36.2)
Married	239 (60.5)
Divorced	11 (2.8)
Widow	1 (0.3)
Single marriage (monogamy)^c^ (*n* = 252)	223 (88.5)
Number of children (Parity, *n* = 252)^c^	
0	41 (16.3)
1–3	166 (65..9)
> 3	31 (12.3)
Any history of self-breast cancer^c^	9 (2.3)
First-degree relatives’ history of breast cancer^c^	40 (10.1)
Second degree relatives or friend’s history of breast cancer^c^	64 (16.2)

^a^Mean (Standard Deviation-SD)/Median (Interquartile Range-IQR)

^b^Frequency (percentage)

^c^Data is missing in participants’ age (*n* = 30), years of experience (24), age at marriage (22), number of pregnancies (11), hospital/Center/department (4), level of education (2), marital status (1), single marriage (22), number of children (14), number of abortions (23), one or more stillbirths (23), any history of self-breast cancer (41), first-degree relatives’ history of breast cancer (41), second degree relatives or friend’s history of breast cancer (54). In the calculation of percentages (%), the denominators include missing observations

### Participants’ knowledge about breast Cancer

The knowledge score achieved in this study is very low; the median score of (range) = 1(0–5). When ranked in order, the 75th percentile is =5 (it means knowledge of only 5 items on the scale). Therefore, in this study, we considered a score of [5–14] as fair and a score of (> = 15) as good. The total score was therefore categorized as poor knowledge (score of 0–4), fair knowledge (score of 5–14), and good knowledge (score of 15–30). About 14 to 26% of the participants responded ‘Yes’ to the following potential risk factors for breast cancer: high-fat diet, working-class women, alcohol consumption, first child at a late age, early onset of menarche, late menopause, obesity, and larger breast (Table 2).

**Table 2:** Participants’ Knowledge About Breast Cancer

Questionnaire items for assessing knowledge about breast cancer	Frequency (%)^a^	95% CI ^b^
Potential risk factors for developing breast cancer:		
R1: Increasing age^d^	18 (4.6)	2.5–6.6
R2: Positive family history^d^	7 (1.8)	0.5–3.1
R3: High-fat diet^d^	58 (14.7)	11.2–18.2
R4: Smoking^d^	36 (9.1)	6.3–12.0
R5: Race/ethnicity^d^	51 (12.9)	9.6–16.2
R6: Working class women^d^	74 (18.7)	14.9–22.6
R7: Alcohol consumption^d^	62 (15.7)	12.1–19.3
R8: First child at late age^d^	92 (23.3)	19.1.0–27.5
R9: Early onset of menarche^d^	101 (25.6)	21.2–29.9
R10: Late menopause^d^	103 (26.1)	21.7–30.4
R11: Stress^d^	46 (11.6)	8.5–14.8
R12: Obesity^d^	57 (14.4)	10.9–17.9
R13: Larger breast^d^	94 (23.8)	19.6–28.0
Sign and symptoms which you think are related to breast cancer:		
SS1: Lump in the breast^d^	7 (1.8)	0.5–3.1
SS2: Discharge from the beast^d^	6 (1.5)	0.3–2.7
SS3: Pain or soreness in the breast^d^	7 (1.8)	0.5–3.1
SS4: Change in the size of the breast^d^	13 (3.3)	1.5–5.1
SS5: Discoloration /dimpling of the breast^d^	7 (1.8)	0.5–3.1
SS6: Ulceration of the breast^d^	15 (3.8)	1.9–5.7
SS7: Weight loss^d^	40 (10.1)	7.1–13.1
SS8: Changes in the shape of the breast^d^	16 (4.1)	2.1–6.0
SS9: Inversion/pulling in of nipple^d^	35 (8.9)	6.0–11.8
SS10: Swelling or enlargement of the breast^d^	16 (4.1)	2.1–6.0
SS11: Lump under armpit^d^	18 (4.6)	2.7–8.4
SS12: Scaling/dry skin in nipple region^d^	49 (12.4)	9.1–15.7
Methods of diagnosis:		
M1: Pathological examination of breast tissue by using FNAC (Fine Needle Aspiration Cytology)^d^	32 (8.1)	5.4–10.8
M2: Self-Breast Examination^d^	6 (1.5)	0.3–2.7
M3: CBE by doctor^d^	4 (1.0)	0.0–2.0
M4: Mammography^d^	3 (0.8)	0.0–1.6
M5: Ultrasound^d^	21 (5.3)	3.1–7.5
Median (IQR) total score (TS^d^) for the knowledge scale^c^	1 (0–5)	
Level of knowledge based on the total score:		
Poor (Score of 0–4)	281 (71.1)	
Fair (Score of 5–14)	104 (26.3)	
Good (Score of 15–30)	5 (1.3)	

^a^Frequencies and percentage (%) for the “yes” responses, % are computed with missing observations included in the denominator

^b^95% Confidence intervals in column 3 for the percentages (%) in column 2

^c^Responses to each item described in column 1 were recoded as (Yes = 1, No or don’t know = 0) and the total score (0–30) for the knowledge scale was computed, the median total score (interquartile range-IQR) was presented in the table

^d^Data is missing in R1 for (8 participants), R2 (7), R3 (18), R4 (13), R5 (15), R6 (22), R7 (13), R8 (17), R9 (21), R10 (18), R11 (12), R12 (27), R13 (17); SS1 (8), SS2 (11), SS3 (12), SS4 (9), SS5 (9), SS6 (11), SS7 (13), SS8 (14), SS9 (12), SS10 (9), SS11 (9), SS12 (16); M1 (10), M2 (8), M3 (9), M4 (7), M5 (9); TS (5)

For other risk factors listed in the questionnaire, less than 14% responded ‘Yes’. Under the section of signs and symptoms of breast cancer, 49 (12.4%) participants agreed that scaling/dry skin in the nipple region could be a sign of breast cancer and 40 (10.1%) participants knew that weight loss could also be a sign of breast cancer. Less than 10% of the participants responded ‘Yes’ to rest of the signs/symptoms. Similarly, a lower rate (< 10%) was observed for the methods of diagnosis of breast cancer. The median (IQR) total score of knowledge about breast cancer was 1 (0–5), and only 5 (1.3%) participants appeared to have good level of knowledge (score: 15–30), while 104 (26.3%) scored fair level knowledge (score:5–14).

Slightly higher proportion of the fair (score: 5–14) knowledge was achieved by other allied health workers 27 (38.6%) comparing to 13 (21.7%) by the physician, and 64 (24.6%) by the nurses, however, this was not significant statistically (*p* = 0.113). When compared, there was no statistically significant difference of proportions of the fair (score: 5–14) knowledge among female physicians working under different specialties at KFMC (*p* = 0.183).

### Participants’ attitudes toward breast Cancer screening and self-examination

Table 3 shows the responses on the statements for attitudes toward breast cancer screening and self-examination. Only 20 (5.1%) participants believed that any woman is at risk of breast cancer and 37 (9.4%) believed that breast cancer can be prevented (Table 3). Also, 53.4% of the participants believed that they could not detect abnormalities in breast by self-examination.

**Table 3:** Participants’ Attitudes Toward Breast Cancer Screening and Self-Examination

Statements for assessing attitudes toward breast cancer, screening and self-examination	Agree n (%)	Neither Agree nor Disagree n (%)	Disagree n (%)
A1: Any woman is at risk for breast cancer^a^	20 (5.1)	24 (6.2)	340 (88.5)
A2: Breast cancer can be prevented^a^	37 (9.4)	60 (15.1)	285 (72.1)
A3: If I examine my breast myself, I cannot detect abnormalities in my breast^a^	203 (53.4)	49 (12.4)	129 (32.7)
A4: There is no reason to examine my breasts^a^	289 (73.2)	17 (4.3)	72 (18.2)
A5: If I knew the benefit of breast self-examination, I would have done it by now^a^	37 (9.4)	23 (5.8)	316 (80.0)
A6: Women prefer female doctor for breast examination^a^	17 (4.3)	28 (7.1)	334 (84.6)
A7: If there is no problem in the breasts, periodic breast examinations by a physician are not required^a^	177 (44.8)	57 (14.4)	149 (37.7)
A8: Early detection methods have no effect on treatment^a^	267 (67.6)	22 (5.6)	93 (23.5)
A9: Personal hygiene decrease breast cancer risk^a^	129 (32.7)	99 (25.1)	143 (36.2)
A10: By early diagnosis of breast cancer, the person will have prolonged life^a^	15 (3.8)	34 (8.6)	335 (84.8)

n (%): Frequencies (percentage) of participants, percentage were computed with missing observations included in the denominator

^a^Data is missing in A1 for (11 participants), A2 (13), A3 (14), A3 (17), A5 (19), A6 (16), A7 (12), A8 (13), A9 (24), and A10 (11)

### Knowledge and practice of breast self-examination

Results for the knowledge and practice of BSE are presented in Table 4. Overall, 370 (93.7%) participants were aware of BSE, and 358 (90.6%) agreed that it is a useful tool for early detection of breast cancer. A total of 336 (85.1%) participants had been taught about BSE and 295 (74.7%) participants reported to be practicing it. Overall, 170 (43.0%) participants chose that BSE should be started from puberty, and 91 (23.0%) chose the age of 20 years to start doing BSE. A total of 317 (80.3%) participants agreed that the best time for BSE is a week after period and 293 (74.2%) participants agreed that BSE should be done monthly. Overall, 362 (91.7%) participants agreed that BSE is a good practice.

**Table 4:** Knowledge and Practice of Breast Self-Examination

Questions/statements for assessing knowledge and practice of BSE	n (%)^a^	95% CI^b^
Q1: Yes- I heard of Breast Self-Examination^c^	370 (93.7)	91.3–96.1
Q2: BSE is a useful tool for early detection of breast cancer^c^	358 (90.6)	87.7–93.5
Q3: Yes- I have been taught about Breast Self-Examination^c^	336 (85.1)	81.5–88.6
Q4: Age at which BSE should be started^c^:		
From birth	4 (1.0)	0.3–2.6
From puberty	170 (43.0)	38.1–48.1
From 20 years	91 (23.0)	19.0–27.5
From 30 years	85 (21.5)	17.1–25.4
After Menopause	3 (0.8)	0.2–2.2
Not sure	34 (8.6)	6.0–11.8
Q5: Time for Breast Self-Examination^c^:		
Daily	8 (2.0)	0.9–4.0
Weekly	23 (5.8)	3.7–8.6
Monthly	293 (74.2)	69.7–78.4
Yearly	32 (8.1)	5.6–11.2
Not Sure	31 (7.6)	5.2–11.0
Q6: What is the best time to do Breast Self-Examination?^c^		
During menstrual flow	23 (5.8)	3.7–8.4
A week after period	317 (80.3)	76.0–84.1
During Pregnancy	2 (0.5)	0.1–1.8
During breastfeeding	1 (0.3)	0.01–1.4
Not sure	46 (11.6)	8.7–15.2
Q7: BSE should be done by^c^		
Doctor	35 (8.9)	6.2–12.1
Trained Nurse	14 (3.5)	2.0–5.9
The Individual	294 (74.4)	69.8–78.7
Combination of the above	35 (8.9)	6.2–12.1
Others	3 (0.8)	0.2–2.2
Not sure	9 (2.3)	1.0–4.3
Q8: BSE is done by^c^:		
Inspecting the breast in the mirror	38 (9.6)	6.9–13.0
Feeling the breast with the hand	150 (38.0)	33.2–43.0
Feeling the armpit with the hand	9 (2.3)	1.0–4.3
Doing Ultrasound of the breast	5 (1.3)	0.4–2.9
Mammography	5 (1.3)	0.4–2.9
Any combination of the above	169 (42.7)	37.8–47.8
Not sure	10 (2.5)	1.2–4.6
Other	2 (0.5)	0.1–1.8
Q9: Action upon abnormality in Breast on Self-Examination^c^:		
Leave it to God and pray	4 (1.0)	0.3–2.6
Do some lab tests	41 (10.4)	7.6–13.8
See a doctor	297 (75.2)	71.0–79.3
Combinations of the above three	36 (9.1)	6.5–12.4
Do nothing	2 (0.5)	0.1–1.8
Others	1 (0.3)	0.01–1.4
Not Sure	9 (2.3)	1.0–4.3
Q10: Benefits of Breast Self-Examination^c^:		
To be familiar with the breast texture	12 (3.0)	1.6–5.2
Early detection of breast cancer	101 (25.5)	21.3–30.2
Detection of any abnormal changes in the breast	143 (36.2)	31.5–41.2
A good breast exercise	4 (1.0)	0.3–2.6
Combinations of the above	122 (30.8)	26.4–35.7
Not Sure	8 (2.0)	0.9–4.0
Q11: Yes- I do practice Breast Self-Examination^c^	295 (74.7)	70.1.0–78.9
Q12: Time for above examination (*n* = 295)^c^:		
Weekly	11 (3.7)	1.9–6.6
Monthly	164 (55.5)	49.7–61.4
Occasionally	93 (31.5)	26.3–37.2
Rarely	24 (8.1)	5.3–11.9
Q13: If no, why not? (*n* = 94)		
I see no reason to do it	25 (26.6)	18.0–36.7
I am afraid of the procedure	26 (27.7)	18.9–37.8
I am afraid of the bad results	11 (11.7)	6.0–20.0
Others	21 (22.3)	14.4–32.0
Q14: Yes- I have discovered abnormality in my breast^c^	59 (14.9)	8.4–23.7
Q15: If answer to the question above is yes, what did you do?^c^ (*n* = 59)		
Leave it to God and pray	6 (10.2)	3.8–20.8
Did some lab. Tests	3 (5.1)	1.1–14.1
Saw a doctor	41 (69.5)	56.1–80.8
Did nothing	2 (3.4)	0.4–11.7
Others	2 (3.4)	0.4–11.7
Q16: Yes- BSE is a good practice^c^	362 (91.7)	88.5–94.2

^a^Frequencies and percentage (%) of participants’ responses, % are computed with missing observations included in the denominator

^b^95% Confidence intervals in column 3 for the percentages (%) in column 2

^c^Data is missing in Q1 for (8 participants), Q2 (7), Q3 (18), Q4 (8), Q5 (8), Q6 (6), Q7 (5), Q8 (7), Q9 (5), Q10 (5), Q11 (5), Q12 (not applicable = 100, missing = 3), Q13 (not applicable = 301, missing = 11), Q14 (15), Q15 (not applicable = 336, missing = 5), Q16 (9)

### Knowledge and practice of clinical breast examination

Results for the knowledge and practice of CBE are presented in Table 5. A total of 345 (87.3%) participants believed that CBE is a useful tool for detection of breast cancer, but only 95 (24.1%) had undergone CBE. Also, 273 (60.0%) respondents chose that a physician should do CBE, 131 (33.2%) believed that mammography should be used in CBE, and 190 (48.1%) agreed that the examination should be conducted at an interval of 1 year.

**Table 5:** Knowledge and Practice of Clinical Breast Examination

Questions/statements for examining knowledge and Practice of CBE	n (%)^a^	95% CI^b^
Q1: Yes- I have heard of CBE^c^	339 (85.8)	84.1–90.9
Q2: Yes- CBE is a useful tool for detection of breast cancer^c^	345 (87.3)	86.1–92.4
Q3: CBE should be done by^c^		
Doctor	273 (60.0)	55.0–64.9
Trained Nurse	31 (7.9)	5.3–11.0
Yourself	47 (11.9)	8.9–15.5
Combinations of the above	49 (12.4)	9.3–16.1
Not sure	21 (5.3)	3.3–8.0
Others	1 (0.3)	0.01–1.4
Q4: CBE is done using^c^		
Ultrasound	24 (6.1)	3.9–8.9
Mammography	131 (33.2)	28.5–38.0
Hand	92 (23.3)	19.2–27.8
Combinations of the above 3	99 (25.1)	20.9–29.6
Others	1 (0.3)	0.01–1.4
Not sure	36 (9.1)	6.5–12.4
Q5: How often CBE should be done^c^		
Daily	9 (2.3)	1.1–4.3
Weekly	10 (2.5)	1.2–4.6
Monthly	91 (23.0)	19.0–27.5
Yearly	190 (48.1)	43.1–53.2
Not sure	83 (21.1)	17.1–25.4
Q6: Yes-I have undergone CBE^c^	95 (24.1)	20.0–28.6
Q7: If yes, time to repeat: (*n* = 95)^c^		
Monthly	23 (24.2)	16.0–34.1
Yearly	53 (55.8)	45.2–66.0.1
After menopause	2 (2.1)	0.3–7.4
Not sure	9 (9.5)	4.4–17.2
Q8: If no, why not? (*n* = 271)^c^		
I see no reason for the test	94 (34.7)	29.0–40.6
I am afraid of the procedure	24 (8.9)	5.8–12.9
I am afraid of the bad results	7 (2.6)	1.0–5.2
I do not know whom to consult for undergoing this test	47 (17.3)	13.0–22.3
Combinations of the above	3 (1.1)	0.2–3.2
Others	25 (9.2)	6.1–13.3

^a^Frequencies and percentage (%) of participants’ responses, % are computed with missing observations included in the denominator

^b^95% Confidence intervals in column 3 for the percentages (%) in column 2

^c^Data is missing in Q1 for (9 participants), Q2 (10), Q3 (9), Q4 (12), Q5 (12), Q6 (6), Q7 (not applicable = 300, missing = 8), Q8 (not applicable = 124, missing = 71)

### Knowledge and use of mammography

Overall, 368 (93.2%) participants had heard about mammography. A total of 287 (72.7%) participants agreed that mammography should be started at 40 years of age and 183 (46.3%) participants believed that mammography should be done every year. Seventy-four (18.7%) participants had undergone mammography (Table 6). Out of these 18.7% women, 59.5% were aged above 41 years while 40.5% were either less than or equal to 41 years of age.

**Table 6:** Knowledge and Use of Mammography

Questions about mammography	n (%)^a^	95% CI ^b^
M1: Yes- I have heard of mammography^c^	368 (93.2)	90.2–95.4
M2: Yes- mammography a useful tool for the early detection of breast cancer^c^	371 (93.9)	91.1–96.1
M3: Age at which mammography should be started:^c^		
From birth	1 (0.3)	0.01–1.4
From puberty	17 (4.3)	2.5–6.8
From 20 years	28 (7.1)	4.8–10.1
From 40 years	287 (72.7)	68.0–77.0
After menopause	18 (4.6)	2.7–7.7
Combinations of the some of the above	3 (0.8)	0.2–2.2
Not sure	33 (8.4)	5.8–11.5
M4: How often should mammography be done?^c^		
Weekly	3 (0.8)	0.2–2.2
Monthly	19 (4.8)	2.9–7.4
Every year	183 (46.3)	41.3–51.4
When a lump is found on BSE or CBE	96 (24.3)	20.2–28.8
Combinations of the above	17 (4.3)	2.5–6.8
Not sure	65 (16.5)	12.9–20.5
M5: Yes- I have done a Mammography^c^	74 (18.7)	15.0–22.9
M6: If no to question above, why not?(*n* = 313)^c^		
Not old enough	104 (33.2)	28.0–38.7
Financial constraint	4 (1.3)	0.3–3.2
Mammography not available	5 (1.6)	0.5–3.7
I see no reason for the test	75 (24.0)	19.3–29.1
I am afraid of the procedure	20 (6.4)	3.9–9.7
I am afraid of the bad results	8 (2.6)	1.1–5.0
I do not know whom to consult for undergoing this test	22 (7.0)	4.6–10.4
Combinations of the above	40 (12.8)	9.3–17.0
Others	20 (6.4)	3.9–9.7
M7: If yes, how often do you go for Mammography? (*n* = 74)^c^		
Monthly	2 (2.7)	0.3–9.4
Yearly	50 (67.6)	55.7–78.0
After menopause	1 (1.4)	0.03–7.3
Not sure	5 (6.8)	2.2–15.1
Others	13 (17.6)	9.7–28.2

^a^Frequencies and percentage (%) of participants’ responses, % are computed with missing observations included in the denominator

^b^95% Confidence intervals in column 3 for the percentages (%) in column 2

^c^Data is missing in M1 for (8 participants), M2 (9), M3 (8), M4 (12), M5 (8), M6 (not applicable = 82, missing = 15), M7 (not applicable = 321, missing = 3),

Under reasons for not undergoing mammography, 104 (33.2%) participants responded that they were not old enough and 75 (24.0%) didn’t believe there was any reason to undergo mammography.

## Discussion

Knowledge and awareness play a vital role in early detection and optimal treatment of breast cancer. The knowledge level of healthcare professionals and their attitudes towards screening methods for breast cancer are important determinants of the practice of these methods by their patients. This study was, therefore, conducted to evaluate the knowledge, attitudes, and practices of breast cancer screening in the female healthcare workers at KFMC. Our cohort demonstrated especially poor knowledge on risk factors, signs and symptoms, and methods of diagnosis. The knowledge related to breast cancer in our cohort appears to be lesser than that found in some other studies [20–22]. Our results for attitudes of participants towards breast cancer screening were also discouraging, which could be due to lack of knowledge in this study population.

With regard to BSE, the results appeared positive with most participants being aware of the importance of BSE. Their knowledge related to BSE was also satisfactory. Also, almost 75% of the participants reported practicing BSE. This is much higher than the rate for BSE seen in some other studies [19, 20, 23]. This is very encouraging indeed and also a little surprising considering the low level of knowledge and attitude in this cohort. The usefulness of breast self-examination as an appropriate method for early breast cancer detection has been debated in the recent past. Whereas, WHO states that there is no evidence of the effect of screening through BSE, although BSE can empower women and it can be used to create awareness some organizations/countries recommend against BSE altogether (e.g. Dutch guidelines), while others still promote it (ACS, Medscape). In KSA, breast self-examination role is important in regions where mammography may not be offered due to socio-cultural reasons. Besides, statistics indicate that 90% of breast lumps are discovered by women themselves. One of the aspects of screening is that women in developing country settings are more aware of the BSE as the information regarding BSE is transmitted more frequently and is readily acceptable than mammography given the specific cultural norms in KSA. Women would prefer to undergo BSE in the privacy of their homes than to reach out to health care services for mammography, which is also embarrassing and uncomfortable procedure. Also, most of the participants in our study had heard of CBE and believed that it is a useful tool. However, only quarter of the participants had undergone CBE. The results were similar for mammography as well with most being aware of mammography as a screening tool but only a few opting for it. Another important reason for the lower number of participants undergoing screening, especially mammography, could be that it is usually recommended after the age of 40 years and the average age of this cohort was younger [24]. However, the low knowledge of breast cancer is of concern and needs to be addressed. Poorly informed healthcare staff could be a concerning barrier in increasing the awareness of general population. Several other studies have been conducted in the KSA to assess the knowledge and practices of breast cancer screening [19–22, 25–27]. The results of these studies were similar with knowledge and attitudes of women towards breast cancer screening below expectation, thus, emphasizing the need for appropriate steps to spread awareness.

As regards to the practice of screening methods, results from studies conducted in other developing countries have not been very encouraging either. An important barrier in other countries is financial constraints [16, 18, 28]. However, this is not a concern in the KSA where healthcare facilities are provided free of cost. Optimal utilization of these services is what needs to be targeted. Thus, proper education of the healthcare staff as well as general population appears to be the single most crucial step required.

Also, reservations that women may have about screening also need to be addressed. Recently, a study (*N* = 816) was conducted by Abdel-Aziz et al. in the Al Hassa region of KSA to evaluate the perceived barriers for breast cancer screening. They found personal fears such as fear of physicians, fear of results, and fear of hospitals as the main barriers for not practicing screening for breast cancer [29]. Being healthcare professionals themselves, such fears were understandably less commonly seen in our cohort. Knowledgeable healthcare professionals with good communication skills and well-planned educational campaigns could make a difference in helping women overcome their fears and hesitations.

In a study (*N* = 500) conducted in five primary healthcare centers in Najran, Saudi Arabia, 57% of the study participants were unaware of mammogram and BSE and 44% were unaware of CBE [20]. Thus, lack of awareness of the methods of screening was an important barrier in the general population in this region of KSA. A quarter of the patients reported not receiving CBE due to unavailability of female doctors. Although this is another important aspect that needs to be addressed however clinical breast examination as method for breast cancer screening should be used only when mammography is unavailable as per the latest recommendations of the Ministry of health in Saudi Arabia [20]. Interestingly, very few participants in our study believed that women prefer female doctors for breast examination. This could be because women may not be able to openly express this reservation to the healthcare staff and therefore healthcare workers are not aware of this fact. Also, it has been found in studies that women who have frequent contact with their physician are more likely to undergo screening further emphasizing the crucial role of healthcare staff. All this implies that some factors affect the rate of breast cancer screening in any community. These include the knowledge and attitude of the healthcare workers themselves, the educational programs for the healthcare workers and for general public, the faith of the women on their clinicians, and the extent of barriers and steps taken to overcome them [29].

One limitation of our study is that it was conducted at one center. Nevertheless, this study provides important insights on the current knowledge and practices of screening methods in female healthcare workers and emphasizes the need for educational programs for the healthcare staff at KFMC. The results also urge other hospitals in the KSA to conduct similar studies to evaluate gaps in the knowledge, attitudes, and practices in their staff. Moreover, we recommend further studies to validate the questionnaire through analysis as the questionnaire was compiled after in-depth review of several articles in the field of study and more extensive studies to be conducted to draw comparison between the differences in health care and non health care workers knowledge and attitude towards screening and practices.

## Conclusion

Overall, the knowledge, attitudes, and practices of the staff related to breast cancer at KFMC were found to be lower than expected. However, the study population had fairly good awareness of the availability and the usefulness of the screening methods. The results from this study, conducted on women healthcare professionals at KFMC, highlight the need for well-planned and comprehensive educational programs for the hospital staff.

Although, the screening tools and resources are available and free of charge in KSA however there is lack of active educational programs and campaigns directed at healthcare workers. Hence, inadequate knowledge about methods of breast cancer screening and their benefits among them could be the reason for lower than expected results of the study. In addition, a nationalized education and screening program in the region, combined with considerations for social and cultural factors needs to be functional.

## Data Availability

The datasets used and/or analyzed during the current study are available from the KFMC research center through the corresponding author on reasonable request.

## Abbreviations

ACS: American Cancer Society
BSE: Breast self-examination
CBE: Clinical breast examination
FNAC: Fine Needle Aspiration Cytology
IQR: Interquartile range
KFMC: King Fahad Medical City
KSA: Kingdom of Saudi Arabia,
SD: Standard deviation

## References

[1] Bray F, Ferlay J, Soerjomataram I, Siegel RL, Torre LA, Jemal A (2018). Global cancer statistics 2018: GLOBOCAN estimates of incidence and mortality worldwide for 36 cancers in 185 countries. CA Cancer J Clin.

[2] Saggu S, Rehman H, Abbas ZK, Ansari AA (2015). Recent incidence and descriptive epidemiological survey of breast cancer in Saudi Arabia. Saudi Med J.

[3] Kingdom of Saudi Arabia Saudi Health Council Saudi Cancer Registry. Cancer Incidence Report Saudi Arabia 2014. Available at: https://nhic.gov.sa/eServices/Documents/2014.pdf. Accessed 19 Nov 2018.

[4] Alotaibi RM, Rezk HR, Juliana CI, Guure C (2018). Breast cancer mortality in Saudi Arabia: Modelling observed and unobserved factors. PLoS One.

[5] Davidson A, Chia S, Olson R, Nichol A, Speers C, Coldman AJ (2013). Stage, treatment and outcomes for patients with breast cancer in British Columbia in 2002: a population-based cohort study. CMAJ Open.

[6] World Health Organisation. Breast cancer - Early diagnosis and screening. Available at: http://www.who.int/cancer/prevention/diagnosis-screening/breast-cancer/en/. Updated in 2018. Accessed 19 Nov 2018.

[7] World Health Organisation. Cancer. Screening and early detection. Available at: http://www.euro.who.int/en/health-topics/noncommunicable-diseases/cancer/policy/screening-and-early-detection. Accessed 19 Nov 2018.

[8] Smith RA, Andrews KS, Brooks D, Fedewa SA, Manassaram-Baptiste D, Saslow D (2017). Cancer screening in the United States, 2017: a review of current American Cancer Society guidelines and current issues in cancer screening. CA Cancer J Clin.

[9] Chouchane L, Boussen H, Sastry KS (2013). Breast cancer in Arab populations: molecular characteristics and disease management implications. Lancet Oncol.

[10] Use of Screening Strategies for Detection of Breast Cancer. Kingdom of Saudi Arabia: MoH, The Saudi Center for EBHC Clinical Practice Guideline; April 2014. Available from: https://www.moh.gov.sa/depten/TCP/Documents/88.%20Breast%20Cancer%20-%20Use%20of%20Screening%20Strategies%20for%20the%20Detection%20of%20Breast%20Cancer.pdf. Accessed 19 Dec 2018.

[11] El Bcheraoui C, Basulaiman M, Wilson S, Daoud F, Tuffaha M, AlMazroa MA (2015). Breast cancer screening in Saudi Arabia: free but almost no takers. PLoS One.

[12] Coleman EA, Lord J, Heard J, Coon S, Cantrell M, Mohrmann C (2003). The Delta project: increasing breast cancer screening among rural minority and older women by targeting rural healthcare providers. Oncol Nurs Forum.

[13] Mandal R, Basu P. Cancer screening and early diagnosis in low and middle income countries. Bundesgesundheitsblatt-Gesundheitsforschung-Gesundheitsschutz. 2018;61(12):1505–12. 10.1007/s00103-018-2833-9 30353287

[14] Akpinar YY, Baykan Z, Nacar M, Gun I, Cetinkaya F (2011). Knowledge, attitude about breast cancer and practice of breast cancer screening among female health care professionals: a study from Turkey. Asian Pac J Cancer Prev.

[15] Othman A, Ahram M, Al-Tarawneh MR, Shahrouri M (2015). Knowledge, attitudes and practices of breast cancer screening among women in Jordan. Health Care Women Int.

[16] Naqvi AA, Zehra F, Ahmad R, Ahmad R, Ahmad N, Yazdani N (2018). Awareness, knowledge and attitude towards breast cancer, breast screening and early detection techniques among women in Pakistan. J Pak Med Assoc.

[17] Ibrahim NA, Odusanya OO (2009). Knowledge of risk factors, beliefs and practices of female healthcare professionals towards breast cancer in a tertiary institution in Lagos, Nigeria. BMC Cancer.

[18] Ramakant P, Singh KR, Jaiswal S, Singh S, Ranjan P, Rana C (2018). A survey on breast Cancer awareness among medical, paramedical, and general population in North India using self-designed questionnaire: a prospective study. Indian J Surg Oncol.

[19] Mahfouz AA, Hassanein MH, Nahar S, Farheen A, Gaballah II, Mohamed A (2013). Breast cancer knowledge and related behaviors among women in Abha City, southwestern Saudi Arabia. J Cancer Educ.

[20] Alshahrani M, Alhammam SY, Al Munyif HA, Alwadei AM, Alwadei AM, Alzamanan SS, Aljohani NS. Knowledge, Attitudes, and Practices of Breast Cancer Screening Methods Among Female Patients in Primary Healthcare Centers in Najran, Saudi Arabia. J Cancer Educ. 2018:1–6. 10.1007/s13187-018-1423-8 PMC6882780 30191519

[21] Dandash KF, Al-Mohaimeed A (2007). Knowledge, attitudes, and practices surrounding breast cancer and screening in female teachers of Buraidah, Saudi Arabia. Int J Health Sci (Qassim).

[22] Jahan S, Al-Saigul AM, Abdelgadir MH (2006). Breast cancer. Knowledge, attitudes and practices of breast self examination among women in Qassim region of Saudi Arabia. Saudi Med J.

[23] Ravichandran K, Al-Hamdan NA, Mohamed G (2011). Knowledge, attitude, and behavior among Saudis toward cancer preventive practice. J Family Community Med.

[24] Bevers TB, Helvie M, Bonaccio E, Calhoun KE, Daly MB, Farrar WB (2018). Breast Cancer screening and diagnosis, version 3.2018, NCCN clinical practice guidelines in oncology. J Natl Compr Cancer Netw.

[25] Milaat WA (2000). Knowledge of secondary-school female students on breast cancer and breast self-examination in Jeddah, Saudi Arabia. East Mediterr Health J.

[26] Hussein DM, Alorf SH, Al-Sogaih YS, Alorf SH, Alaskar RS, Al-Mahana AM (2013). Breast cancer awareness and breast self-examination in northern Saudi Arabia. A preliminary survey. Saudi Med J.

[27] Al-Wassia RK, Farsi NJ, Merdad LA, Hagi SK (2017). Patterns, knowledge, and barriers of mammography use among women in Saudi Arabia. Saudi Med J.

[28] Mamdouh HM, El-Mansy H, Kharboush IF, Ismail HM, Tawfik MM, El-Baky MA (2014). Barriers to breast cancer screening among a sample of Egyptian females. J Family Community Med.

[29] Abdel-Aziz SB, Amin TT, Al-Gadeeb MB, Alhassar AI, Al-Ramadan A, Al-Helal M (2018). Perceived barriers to breast cancer screening among Saudi women at primary care setting. J Prev Med Hyg.

